# Machine learning profiles of cardiovascular risk in patients with diabetes mellitus: the Silesia Diabetes-Heart Project

**DOI:** 10.1186/s12933-023-01938-w

**Published:** 2023-08-24

**Authors:** Hanna Kwiendacz, Agata M. Wijata, Jakub Nalepa, Julia Piaśnik, Justyna Kulpa, Mikołaj Herba, Sylwia Boczek, Kamil Kegler, Mirela Hendel, Krzysztof Irlik, Janusz Gumprecht, Gregory Y. H. Lip, Katarzyna Nabrdalik

**Affiliations:** 1 Department of Internal Medicine, Diabetology and Nephrology, Faculty of Medical Sciences in Zabrze, Medical University of Silesia, Katowice, Poland; 2 Faculty of Biomedical Engineering, Silesian University of Technology, Zabrze, Poland; 3 Department of Algorithmics and Software, Silesian University of Technology, Gliwice, Poland; 4 Students’ Scientific Association by the Department of Internal Medicine, Diabetology and Nephrology in Zabrze, Faculty of Medical Sciences in Zabrze, Medical University of Silesia, Katowice, Poland; 5 Liverpool Centre for Cardiovascular Science at University of Liverpool, Liverpool John Moores University and Liverpool Heart & Chest Hospital, Liverpool, UK; 6 Danish Center for Health Services Research, Department of Clinical Medicine, Aalborg University, Aalborg, Denmark

**Keywords:** Diabetes mellitus, Machine learning, Cardiovascular disease, Prediction model, Michigan neuropathy screening instrument

## Abstract

**Aims:**

As cardiovascular disease (CVD) is a leading cause of death for patients with diabetes mellitus (DM), we aimed to find important factors that predict cardiovascular (CV) risk using a machine learning (ML) approach.

**Methods and results:**

We performed a single center, observational study in a cohort of 238 DM patients (mean age ± SD 52.15 ± 17.27 years, 54% female) as a part of the Silesia Diabetes-Heart Project. Having gathered patients’ medical history, demographic data, laboratory test results, results from the Michigan Neuropathy Screening Instrument (assessing diabetic peripheral neuropathy) and Ewing’s battery examination (determining the presence of cardiovascular autonomic neuropathy), we managed use a ML approach to predict the occurrence of overt CVD on the basis of five most discriminative predictors with the area under the receiver operating characteristic curve of 0.86 (95% CI 0.80–0.91). Those features included the presence of past or current foot ulceration, age, the treatment with beta-blocker (BB) and angiotensin converting enzyme inhibitor (ACEi). On the basis of the aforementioned parameters, unsupervised clustering identified different CV risk groups. The highest CV risk was determined for the eldest patients treated in large extent with ACEi but not BB and having current foot ulceration, and for slightly younger individuals treated extensively with both above-mentioned drugs, with relatively small percentage of diabetic ulceration.

**Conclusions:**

Using a ML approach in a prospective cohort of patients with DM, we identified important factors that predicted CV risk. If a patient was treated with ACEi or BB, is older and has/had a foot ulcer, this strongly predicts that he/she is at high risk of having overt CVD.

## Introduction

Every five seconds, one person in the world dies due to diabetes mellitus (DM) and its complications [1]. Cardiovascular disease (CVD) takes the greatest toll here, as it is a leading cause of death for both patients with type 1 diabetes mellitus (T1DM) and type 2 diabetes mellitus (T2DM) [2]. DM itself doubles the risk of coronary artery disease, ischemic stroke, and vascular deaths, independently of other risk factors [3]. It is estimated that patients with DM and CVD and/or chronic kidney disease as well as those having at least three CVD risk factors or with DM duration over 20 years, are at a very high risk, with the 10-year risk of CVD death exceeding 10% [4]. Hence, it is clinically important to identify patients with the highest CVD risk to implement preventive procedures against cardiovascular (CV) events which may otherwise lead to death.

Although several calculators assessing CVD have been developed [5, 6], they are usually validated in a general population and do not accurately assess the risk among patients with DM [7, 8]. Therefore, dedicated risk models are proposed for this group [9], but yet it remains unclear which one is optimal [10]. Moreover, the risk assessment models are not personalized, hence they do not exploit patient-specific granular information that could be crucial in understating the risk of a particular person. Furthermore, they utilize classical statistical methods which commonly show only a potential association for the population studied but cannot necessarily predict the individual’s risk [8]. That is why personalized prediction tools benefiting from data-driven machine learning (ML) approaches which can not only indicate the associations but also anticipate the future risk are of paramount practical importance and attract research much attention.

ML techniques can help uncover new clinical features and relationships between them that are pivotal while identifying high-risk patients, therefore, new risk factors that were not previously taken into consideration in traditional models can emerge, especially in multimorbid high risk patients [11–13]. Lately, unsupervised ML clustering has been successful in the detection of coronary artery atherosclerosis among T2DM patients, whereby on the basis of coronary computed tomography angiography, the algorithm was able to distinguish different plaque types and extents of coronary artery stenosis [14]. Moreover, predicting CVD events with ML models is more effective and offers more accurate estimations in comparison with traditional risk calculators [11, 12].

In our recent work, we demonstrated that ML can be utilized to identify new risk factors for predicting CVD in patients with metabolic-associated fatty liver disease [15], and for predicting cardiovascular events among patients with DM [16]. However, these experiments did not include any subgroup analysis of DM patients with associated diabetic peripheral neuropathy (DPN) or cardiovascular autonomic neuropathy (CAN) and those without these conditions which could be important factors in understanding a personal CVD risk, since those microvascular complications are associated with CVD [17, 18]. To tackle this research gap, we determine the risk of CVD among patients with DM in relation to the presence of diabetic neuropathy, with the use of ML approaches.

Significant efforts are put nowadays into designing precision medicine techniques, where accurate phenotyping of the patients might help in the diagnosis and prognosis of the disease, provide ‘real time’ risk assessment and improve management via implementation of tailored approaches for individuals [19, 20]. Following this research pathway, we aimed to precisely classify DM patients using prospectively collected granular individual data, with a special emphasis put on the DPN and CAN examination, according to the CV risk, and to profile their phenotypes which might be implemented in everyday clinical practice.

## Patients and methods

We performed a single center, observational study in a cohort of T1DM and T2DM consecutive patients hospitalized in the Department of Internal Medicine and Diabetology in Zabrze, Poland, and patients from the Outpatient Diabetology Clinics in the Silesia Region, Poland, from October 6, 2021 to December 15, 2022. This is a part of the Silesia Diabetes-Heart Project (ClinicalTrials.gov Identifier: NCT05626413).

### Inclusion and exclusion criteria

The inclusion criteria for the study were as follows: age ≥ 18 years and ≤ 85 years, T1DM for at least 5 years or T2DM of any duration. The exclusion criteria included: the lack of consent for participation in the study, other than T1DM and T2DM types of diabetes, any severe and acute illness, disabled and bedridden patients, solid organ transplant, other than diabetes previously diagnosed causes of neuropathy, pregnancy, alcoholism, severe hypoglycemia in the past 24 h, an estimated glomerular filtration rate (eGFR) < 30 ml/min/1.73 m^2^, and the proliferative retinopathy.

### Ethics committee consent

The study was performed in accordance with the ethical principles of the Declaration of Helsinki and approved by the Ethics Committee of the Medical University of Silesia (KNW/0022/KB1/10/17). A written informed consent to participate in the study was obtained from all patients enrolled into this study.

### Medical history

Following written informed consent, we collected patients’ demographic and clinical data including detailed documented medical history including pharmacotherapy. The presence of CVD was defined as at least one of the following: coronary artery disease, history of coronary revascularization, percutaneous cardiac intervention or coronary artery bypass grafting, atrial fibrillation, history of myocardial infraction or stroke/transient ischemic attack (TIA), carotid atherosclerosis (defined as carotid stenosis of at least 50% in diameter [21]) and/or lower limb atherosclerosis.

### Anthropometric measurements

Every participant had anthropometric measurements performed with the use of the weight with height gauge SECA 799, which included the measurement of height (in meters) and body weight (in kilograms). The body mass index (BMI) was calculated by dividing the weight in kilograms by height in meters squared (kg/m^2^).

### Laboratory test results

On the day of the site visit, fasting venous blood was drawn, and morning urine spots from previous 3 days were collected (on the day of the informed consent signing, each patient has been instructed about the proper management of urine void). The following blood and urine biochemical parameters were assessed: hemoglobin A1c (HbA1c), serum creatinine concentration, lipid profile, and urinary albumin to creatinine ratio (UACR). HbA1c was measured using a high-performance liquid chromatography method (HPLC), and the results were expressed in the National Glycohemoglobin Standardization Program/Diabetes Control and Complications trial units [22]. Serum creatinine concentration was measured using the Jaffe’s method [23], and estimated glomerular filtration rate was calculated on the basis of the Chronic Kidney Disease Epidemiology Collaboration (CKD-EPI) formula [24]. Enzymatic methods were used to measure cholesterol and triglycerides concentration, whereas the concentration of the low-density lipoprotein cholesterol was calculated through the Friedewald formula [25]. The UACR was estimated using immunoturbidimetric methods and expressed in mg/g creatinine [26].

### The diagnosis of diabetic peripheral neuropathy

We used The Michigan Neuropathy Screening Instrument (MNSI) [27] which is designed for patients with diabetes to assess the presence of DPN. It was used for the first time in 1994 and since then it has been widely adopted. The MNSI consists of two separate parts: a 15-item questionnaire and a lower extremity examination. The 15-item questionnaire was completed independently by each patient participating in the study. Positive responses to all questions 1–15 (except for questions 7 and 13) count as 1 point, while to questions 7 and 13 negative responses count as 1 point. All the points are summed up to obtain the final score, and the score ≥ 7 points indicate DPN. The second component of MNSI is a foot examination performed by healthcare professionals. Each foot is inspected for appearance (0–normal; 1–abnormal), ulcerations (0–absent; 1–present), Achilles tendon reflex (0–present; 0.5–present with reinforcement; 1–absent), and vibration sensation tests with a 128-Hz tuning-fork (0–correct; 0.5–reduced; 1–absent). Each foot is assessed separately and the final score is a sum of all the examined aspects. A score ≥ 2.5 is considered abnormal [27]. If a patient had ulceration of foot, we used the term the diabetic foot syndrome [28, 29] and we divided it into the current ulceration (during the MNSI examination) and a healed one (medical history collection).

### The diagnosis of cardiovascular autonomic neuropathy

For the diagnosis of CAN, we used DiCAN (Diabetic Cardiac Autonomic Neuropathy, Medicore) which exploits an Ewing battery. In the diagnosis of CAN, the American Diabetes Association recommends the use of Ewing’s tests (the so-called Ewing battery developed in the 1970s) [30], including five non-invasive cardiovascular reflex tests, i.e., (1) Heart rate (HR) response to deep breathing; (2) HR response to standing up; (3) Blood pressure response to standing up; (4) Valsalva maneuver; (5) Blood pressure response to sustained handgrip, to assess autonomic functions [30, 31]. The first two tests measure parasympathetic function (primarily the ability of the vagus nerve to slow down HR during heart rate-increasing procedures), while the third and fifth tests measure sympathetic function (blood pressure fluctuations) using baroreceptors. In contrast, the Valsalva maneuver has both parasympathetic and sympathetic components [30]. Subsequently, on the basis of the abovementioned test results, the DiCAN device suggests a diagnosis (normal, early involvement, severe involvement, definite involvement, atypical pattern).

### Quality of life

Since diabetic neuropathy may influence quality of life, we tested the patients with the SF-36 questionnaire [32] which is the RAND (research and development) Health Survey (Version 1.0) consisting of 36 items and covering 8 concepts of health: physical functioning, role limitations due to physical health problems, role limitations due to personal or emotional problems, energy/fatigue, emotional well-being, social functioning, bodily pain, general health. In addition, it also includes a separate item—the health change which indicates a perceived change in health status [32] (Table 1).

**Table 1:** Clinical patient characteristics

Parameter	Patients without CVD (n = 185)	Patients with CVD (n = 53)	*p*-value
Demographic parameters			
Men, n%	88 (47.57%)	22 (41.51%)	0.435
Age (years)	**48.32 ± 17.26**	**65.51 ± 8.51**	** < 0.0001**
Clinical parameters			
*Diabetes-related*			
BMI (kg/m^2^)	28.48 ± 6.17	30.86 ± 5.29	0.003
Duration of diabetes (years)	10.76 ± 8.48	13.79 ± 10.93	0.078
Type of diabetes (% of type 1)	1.63 ± 0.48	1.91 ± 0.30	< 0.001
*Concomitant diseases*			
Arterial hypertension	92 (49.73%)	48 (90.57%)	< 0.001
Chronic kidney disease	78 (42.16%)	32 (60.38%)	0.019
Healed foot ulceration	**4 (2.16%)**	**3 (5.66%)**	**0.184**
Diabetic peripheral neuropathy	11 (5.95%)	3 (5.66%)	0.938
Diabetic retinopathy	16 (8.65%)	7 (13.21%)	0.322
Laboratory parameters			
eGFR (ml/min/1.73m^2^)	91.52 ± 22.48	74.50 ± 19.56	< 0.0001
HbA1c (%)	8.66 ± 2.19	8.61 ± 2.06	0.958
High density lipoprotein	1.50 ± 0.49	1.40 ± 0.47	0.196
Total cholesterol (mmol/l)	4.72 ± 1.17	4.84 ± 1.51	0.989
Triglycerides (mmol/l)	1.62 ± 1.08	1.78 ± 0.91	0.117
Pharmacotherapy			
ACEi	**49 (26.49%)**	**31 (58.49%)**	** < 0.001**
Alpha-lipoic acid	1 (0.54%)	2 (3.77%)	0.063
Antidepressants	10 (5.41%)	6 (11.32%)	0.129
Antiepileptic drugs	7 (3.78%)	5 (9.43%)	0.097
ARB	23 (12.43%)	9 (16.98%)	0.392
ASA	22 (11.89%)	34 (64.15%)	< 0.0001
Beta-blocker	**46 (24.86%)**	**37 (69.81%)**	** < 0.0001**
GLP-1 RA	10 (5.41%)	6 (11.32%)	0.129
Insulin	122 (65.95%)	35 (66.04%)	0.990
Metformin	96 (51.89%)	36 (67.92%)	0.038
NOAC	0 (0.00%)	3 (5.66%)	0.001
SGLT-2i	31 (16.76%)	12 (22.64%)	0.326
Statin	53 (28.65%)	37 (69.81%)	< 0.0001
Type SGLT-2i	0.37 ± 0.86	0.49 ± 0.97	0.334
VKA	0 (0.00%)	2 (3.77%)	0.008
Diabetic cardiovascular autonomic neuropathy			
Blood pressure analysis in the standing position	0.54 ± 0.72	0.90 ± 0.85	0.005
Blood pressure analysis in the standing position after 1 min	0.50 ± 0.72	0.55 ± 0.78	0.829
Blood pressure analysis in the standing position after 3 min	0.45 ± 0.71	0.55 ± 0.76	0.334
Handgrip test	0.54 ± 0.81	0.50 ± 0.79	0.812
Heart rate variation with deep breathing	0.82 ± 0.93	1.32 ± 0.89	0.001
Test 30:15	0.36 ± 0.65	0.47 ± 0.67	0.166
Valsalva test after 1 minutre	0.34 ± 0.54	0.51 ± 0.71	0.197
Valsalva test after 20 s	0.36 ± 0.69	0.83 ± 0.96	0.002
Ewing’s battery test result	1.79 ± 1.72	2.38 ± 1.50	0.017
Diagnostic parameters of peripheral neuropathy			
Achilles tendon reflex in the left foot	0.03 ± 0.15	0.02 ± 0.14	0.615
Achilles tendon reflex in the right foot	0.02 ± 0.12	0.02 ± 0.14	0.913
Left foot appearance	66 (35.68%)	31 (58.49%)	0.003
Current left foot ulceration	**2 (1.08%)**	**2 (3.77%)**	**0.179**
Right foot appearance	69 (37.30%)	31 (58.49%)	0.006
Current right foot ulceration	4 (2.16%)	3 (5.66%)	0.184
Vibration perception in the left foot	0.05 ± 0.20	0.12 ± 0.29	0.028
Vibration perception in the right foot	0.04 ± 0.17	0.10 ± 0.25	0.013
Peripheral neuropathy score	14 (7.57%)	11 (20.75%)	0.006
Quality of life questionnaire			
SF36 Emotional well being	60.37 ± 16.60	59.55 ± 16.37	0.629
SF36 Energy fatigue	55.00 ± 17.81	52.74 ± 16.69	0.260
SF36 General health	51.80 ± 17.25	46.04 ± 20.29	0.032
SF36 Health change	41.94 ± 23.29	38.68 ± 22.77	0.235
SF36 Pain	71.16 ± 29.17	58.87 ± 28.81	0.004
SF36 Physical functioning	80.55 ± 23.74	59.43 ± 25.58	< 0.0001
SF36 Role limitation due to emotional problems	65.76 ± 39.45	62.26 ± 41.36	0.592
SF36 Role limitation due to physical health	54.37 ± 41.70	42.45 ± 39.40	0.070
SF36 Social functioning	75.34 ± 27.72	75.00 ± 29.32	0.941
MNSI score	3.84 ± 2.86	5.19 ± 3.21	0.007
PDN diagnosis according to MNSI score	37 (20.00%)	19 (35.85%)	0.016

*ACEi* Angiotensin Converting Enzyme Inhibitor, *ARB* Angiotensin II Receptor Blocker, *ASA* Acetylsalicylic Acid, *BMI* Body Mass Index, *GLP-1 RA* Glucagon-like Peptide-1, *HbA1c* Hemoglobin A1c, *HDL-C* High Density Lipoprotein Cholesterol, *PDN* Peripheral Diabetic Neuropathy, *SGLT-2i* Sodium-Glucose Cotransporter-2, *NOAC* Novel Oral Anticoagulants, *VKA* Vitamin K Anticoagulant

For each parameter (if applicable), we report its mean ± standard deviation (SD), whereas for each binary parameter, the total number of ones and the percentage of ones are given. The p-values were calculated using either Mann–Whitney *U*-test or *χ*^2^ test as appropriate.

The most discriminative features are rendered in bold

### Predicting cardiovascular disease using machine learning

The prediction of the occurrence of a CVD for a patient with diabetes was based on demographic data (2 parameters), clinical data (diabetes-related: 3 parameters and concomitant diseases: 5 parameters), laboratory data (5 parameters), data regarding medications (15 parameters), Ewing’s battery test results (9 parameters), MNSI results (9 parameters) and quality of life (SF-36) questionnaire (11 parameters). In total, 59 features were evaluated (Table 1), with the average number of missing values of 1.42%. Prior to the implementation of feature selection and CVD prediction mechanisms, the missing data was imputed using factorial analysis [33]. The most discriminative predictors were selected using a *χ*^2^ test following a Monte Carlo approach with 1000 repetitions to ensure the stability of the selected features. In each Monte Carlo iteration, we randomly sampled 80% of patients (with overlaps) for whom the most discriminative predictors were selected by picking the features with p < 0.05 obtained by the *χ*^2^ test. Finally, five of the most frequently selected predictors (within 1000 independent repetitions) were considered the most discriminative. It is of note that exploiting the four most frequently picked features leads to statistically significantly worse classification results obtained by the multiple logistic regression (MLR) model (Wilcoxon matched-pairs signed rank test, p < 0.0001), whereas using the six most frequently selected predictors leads to over-fitting of MLR to the dataset.

Then, the MLR model was fitted using the selected discriminators, and the optimal cut-point value was extracted from the receiver operating characteristic curve (ROC) using the Index of Union approach [34]. We also performed unsupervised hierarchical clustering of all patients based on the selected features [35]—the number of groups (clusters) was determined in two ways: (*i*) by considering the binary division into two clusters (we hypothesize that the patients can be grouped into those at high- and low- risk of having CVD), and (*ii*) by determining the optimal number of clusters using the Calinski-Harabasz qualitative criterion [36]. In (*ii*), the cohort of patients may be clustered into a larger number of groups (depending on the patients’ characteristics), potentially corresponding to different patient profiles. To evaluate the classification performance, we report sensitivity, specificity, and the percentage of correctly classified (CC) high- and low-risk patients, i.e., with and without CVD. For the MLR model, the ROC curves, alongside the area under those curves (AUC) were determined. Decision curve analysis (DCA) was used to assess the clinical utility of the model.

The MATLAB R2022b environment was used for feature selection, hierarchical clustering and visualization of results, whereas GraphPad Prism 9.4.1 was exploited for MLR. The visualization of multidimensional feature spaces was realized using t-distributed stochastic neighbor embedding (t-SNE) [37].

## Results

**Fig. 1:**
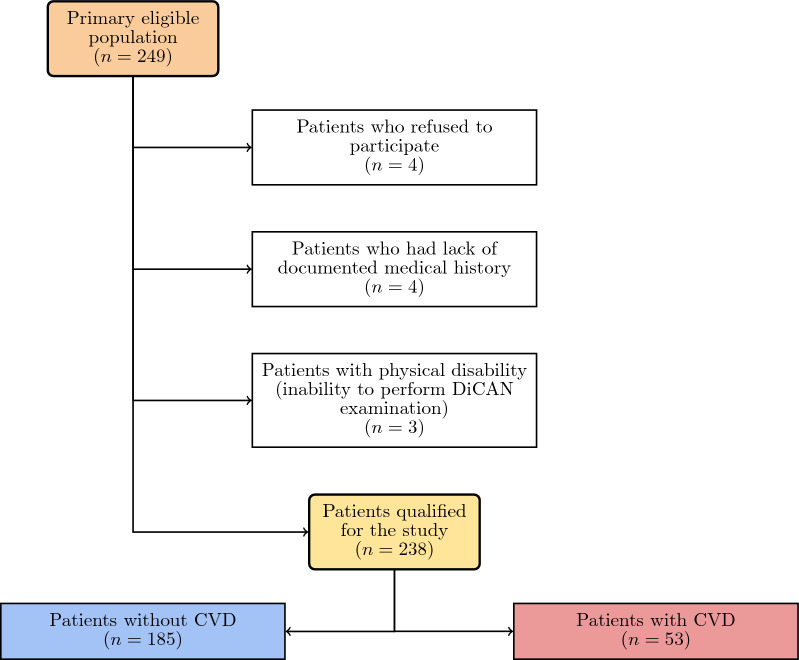
Patient flowchart

A group of 249 patients formed the primary eligible population (Fig. 1), of which 238 (mean age ± SD 52.15 ± 17.27 years, 54% women) were qualified for medical examinations and were included in this study. The reasons for non-completion are presented in Fig. 1. Of the patients, 31% had T1DM and 69% had T2DM. CVD was reported in 53/238 (22%) patients.

Feature selection led to obtaining the five most discriminative predictors: beta-blocker (BB) (selected in 965/1000 Monte Carlo iterations), age (868/1000), current left foot ulceration (627/1000), angiotensin converting enzyme inhibitors (ACEi) (567/1000) and healed foot ulceration (558/1000) (Table 1). The classification performance of the MLR model for determining high-risk patients is quantified in Table 2**.**

**Table 2:** Cardiovascular event prediction results based on the most discriminative [5] features using the multiple logistic regression model and hierarchical clustering

ML approach	Sensitivity	Specificity	CC with event, %	CC without event, %	CC All, %
Multiple logistic regression	0.83	**0.74**	83.02	**73.51**	**75.63**
Hierarchical clustering	**1.00**	0.38	**100.00**	37.84	51.68

The best metrics are boldfaced

**Fig. 2:**
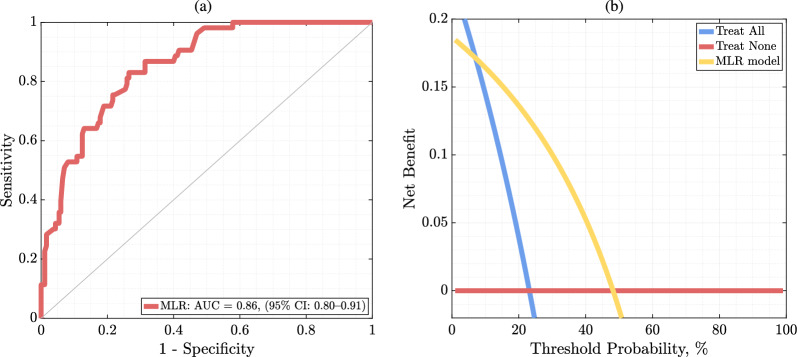
ROC curve and Decision Curve Analysis. **a** The ROC curve obtained using the multiple logistic regression model fitted over the most discriminative [5] patient’s parameters, together with **b** the decision curve analysis presenting clinical utility of the application. In the case of the ROC curve, the 45° curve through the origin shows the classifier's discriminatory ability no better than random sampling

The obtained results indicate that 44/53 (83.02%) high- and 137/185 (73.51%) low-risk patients were correctly identified, thus 181/238 (76.05%) of all patients were correctly classified. For patients with and without diagnosed neuropathy, 16/19 (84.21%) and 28/34 (82.35%) high-risk were respectively correctly identified, with 23/37 (62.16%) and 63/148 (42.57%) low-risk patients were correctly determined, indicating a significantly larger number of false positive high-risk detections in the latter group. The predictive performance of the MLR model is further reflected in its sensitivity and specificity, amounting to 0.83 and 0.74, respectively. The area under the ROC curve for this classifier operating on the five most discriminative features reaches AUC: 0.86 (95% CI 0.80–0.91) (Fig. 2a). The clinical utility of MLR was also determined using DCA (Fig. 2b), which shows that above the 7% probability threshold and below 48%, the model had a higher utility in terms of net benefit than alternative treatment strategies, i.e., treating none or all patients. It is of note that exploiting all (n = 59) predictors in the MLR led to over-fitting, hence to memorizing the dataset, thus the ML model was unable to generalize.

**Fig. 3:**
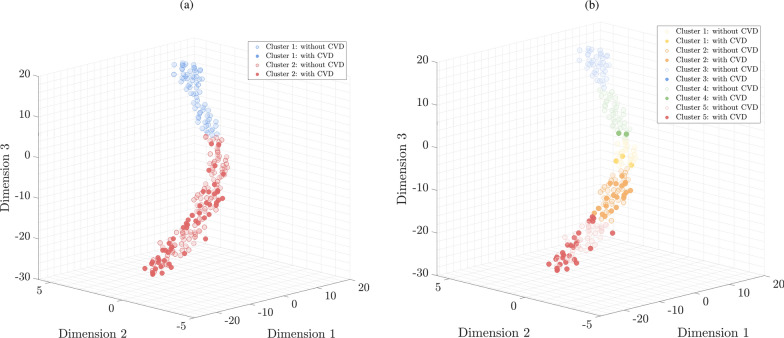
The t-SNE visualization of two hierarchical clustering results obtained for the most discriminative [5] patient parameters with different numbers of clusters: **a** 2 and **b** 5, respectively

Hierarchical clustering of all patients based on the most discriminative patient parameters was performed in two ways. In the first case, patients were divided into two groups (Fig. 3a), as we hypothesize that the patients can be clustered into the high- and low-risk ones. In this case, as for the MLR model, sensitivity, specificity, and the percentages of CC high- and low-risk patients were determined (Table 2). The obtained results indicate high sensitivity of this solution (1.00) with lower specificity compared to the MLR model (0.38 vs. 0.74).

**Fig. 4:**
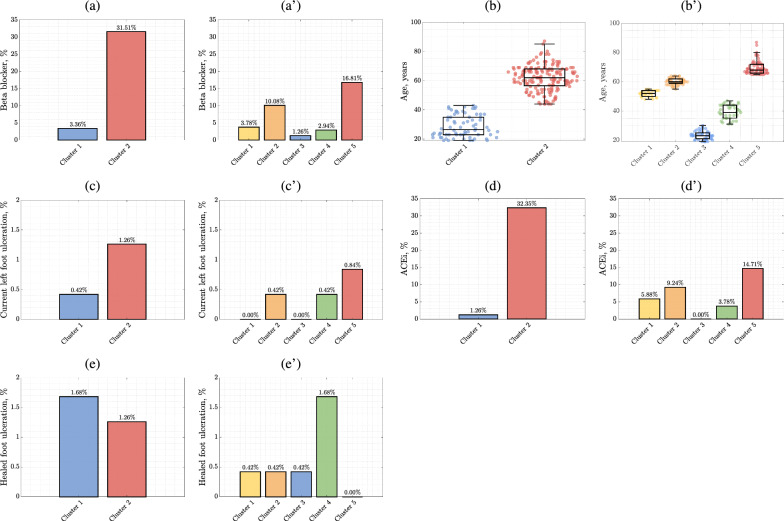
The most discriminative features for individual clusters in (*i*) the binary (two-cluster) approach **a**–**e** and (*ii*) with the optimal number [5] of clusters **a**–**e**′ determined using the Calinski-Harabasz qualitative criterion

From the clinical assessment point of view, it is less costly to classify a low-risk patient as a high-risk one, rather than to miss a patient at a high risk of CVD—here, hierarchical clustering led to correctly classifying all high-risk patients, with an increased number of false positive classifications (i.e., low-risk patients incorrectly classified as being high-risk). The t-SNE visualization of the two-group clustering allows for identifying the group of patients in which there is no risk of CVD (Fig. 3a, cluster 1), and the group at high risk of CVD. Clustering was also carried out for the optimal number of clusters (5 clusters) determined based on the Calinski-Harabasz criterion (Fig. 3b). The cohort, based on the 5 most discriminating features, was clustered into 5 groups for which we observe an increased risk of CVD, reflected in the increasing number of high-risk patients in the following clusters (Fig. 4). The values of the discriminative patients’ parameters for all clusters (in two- and five-group clustering) are summarized in Table 3 and presented in Fig. 4.

**Table 3:** Patient’s parameter values (% yes) for the most discriminative [5] predictors obtained for patients grouped using hierarchical clustering into 2 and 5 clusters

Parameter	Two-group clustering			Five-group clustering					
	Cluster 1 (n = 70)	Cluster 2 (n = 168)	*p*-value	Cluster 1 (n = 28)	Cluster 2 (n = 56)	Cluster 3 (n = 42)	Cluster 4 (n = 39)	Cluster 5 (n = 73)	*p*-value
Beta blocker	8 (11.43%)	75 (44.64%)	< 0.0001	9 (32.15%)	24 (42.86%)	3 (7.14%)	7 (17.95%)	2 (2.74)	< 0.0001
Age	28.79 ± 7.57	61.89 ± 8.70	< 0.0001	51.75 ± 2.15	60.05 ± 2.28	23.26 ± 3.04	39.31 ± 4.80	69.72 ± 4.60	< 0.0001
Current left foot ulceration	1 (1.43%)	3 (1.79%)	0.845	0 (0.00%)	1 (1.79%)	0 (0.00%)	1 (2.56%)	2 (2.74%)	0.759
ACEi	3 (4.28%)	77 (45.83%)	< 0.0001	14 (50.00%)	22 (39.39%)	0 (0.00%)	9 (23.08%)	35 (47.95%)	< 0.0001
Healed foot ulceration	4 (5.71%)	3 (1.78%)	0.102	1 (3.57%)	1 (1.78%)	1 (2.38%)	4 (10.26%)	0 (0.00%)	0.043
CV event	0 (0.00%)	53 (31.54%)	< 0.0001	3 (10.71%)	20 (35.71%)	0 (0.00%)	2 (5.13%)	28 (38.36%)	< 0.0001

The p-values were calculated using Mann–Whitney U-test, *χ*^2^ test, or Kruskal–Wallis tests, where appropriate

## Discussion

The key findings of our study are that we managed to determine five out of 59 most discriminative patients’ parameters (the presence of past or current foot ulceration, age and the treatment with BB and ACEi) which enabled us to identify patients at risk of CVD. On its basis, we clustered individuals with similar phenotypes in order to stratify their CV risk, showing good predictive value (AUC > 0.8) and clinical usefulness on decision curve analysis.

All of the determined parameters are easy to obtain and interpret, therefore they might be used in everyday practice as they are gathered just based on medical history collection and simple foot visual examination. This finding is of utmost importance as dividing individual patients into high- and low-risk personalized strata enables to tailor a proper treatment pathway for an individual, which stays in line with precision medicine principles [38].

Patient’s age was one of the parameters selected by the model, in accordance with existing knowledge [39], given that older the patient becomes, the higher the risk of CVD is. Subsequent features were related to foot ulceration. For many years, the presence of the diabetic foot syndrome increases the mortality rate more than twice in comparison with DM patients without ulcerations [40], and results in diminished 5-year survival (43%) compared to non-DM patients with ulcerations (56%) [41]. Indeed, life expectancy for patients after amputation is comparable to advanced congestive heart failure of aggressive neoplasm disease [42]. Therefore, diabetic foot syndrome is believed to be a proxy for CVD [43]. Additionally, foot ulcerations are known to take part in the development of atherosclerosis leading to coronary artery disease and exacerbations of CVDs [44, 45].

Two out of five most discriminative patients’ parameters are related to pharmacotherapy, as patients with a higher CV risk more often used BB and ACEi than individuals less prone to CVD. Treatment with those drugs, most probably, does not increase the CV risk per se, but reflect the presence of other comorbidities. The two above-mentioned groups of drugs are commonly used, as the first line therapy, in coronary artery disease, heart failure, and hypertension [46], which demonstrate their utility in CV prediction.

Apart from determining the features increasing the CV risk for DM patients, our analysis clustered patients into high and low-risk strata. Previously, cluster analysis was used to interpret data obtained in the Trial Evaluating Cardiovascular Outcomes with Sitagliptin (TECOS [47]) study and Exenatide Study of Cardiovascular Event Lowering (EXCEL [48]) trial where four distinct phenotypes of patients (differing in terms of CV outcomes) were identified. The highest incidence rate of composite CV outcome was noticed among patients who had the highest mean age (confirmed also in our study), and were predominantly Caucasian males, with the highest median UACR and the lowest eGFR with a prior history of heart failure [49].

In our study, patients were divided into two and five clusters. The first division (2 clusters) was based on the hypothesis that we can infer two clusters of high- and low-risk patients, while the second one (5 clusters) was the automatically determined optimal number of clusters for the analyzed cohort of patients. The two-group clustering revealed that the group of high-risk patients contained the individuals who were older, treated with ACEi and BB, and had past or current foot ulceration. This grouping enabled to correctly classify all of the patients with CVD, which is its great strength, as none of the high-risk patients remained undiagnosed. On the other hand, in a group of individuals without overt CVD, there were some false positive indications, which might be costly considering health care expenditures.

Taking into consideration the five-group clustering, two groups (i.e., clusters 2 and 5) appeared to be of high CV risk—the oldest patients were treated in large extent with ACEi but not BB and having current foot ulceration (cluster 5 in Fig. 3), and slightly younger individuals treated with both of the above-mentioned drugs, with a relatively small percentage of diabetic foot syndrome (cluster 2 in Fig. 3). Using both ACEi and BB probably indicates multimorbidity of the examined patients, while the sole use of ACEi might be used due to its nephroprotective effect as its recommendation in the DM management guidelines [50]. On the basis of our results, we can profile patients’ CV risk and predict that the highest probability of having CVD is associated with clusters 2 or 5. On the other hand, the youngest patients mostly without the concomitant treatment and low percentage of diabetic foot syndrome (cluster 3 in Fig. 3) might be classified as relatively safe from the CV point of view.

### Limitations

We are aware of the limitations of our study. This was a single center study, therefore, its outcomes should be validated over a larger and more heterogeneous population of patients with diabetes to further robustify our findings. Moreover, we were unable to analyze UACR due to high number of missing values (14.29% of all patients did not have the UACR parameter calculated), which was one of the parameters important in cluster analysis of patients from EXCEL and TECOS trials [49]. Utilizing other algorithms for imputing missing values, also for those parameters with relatively large percentage of missing data points, may indeed constitute an interesting research pathway to allow for including such parameters in the predictive ML models [51, 52]. Although we exploited the MLR models for determining high-risk patients, utilizing other data-driven techniques, built upon both classic [53–55] and deep [56] machine learning approaches, may not only help improve the classification performance of the system, but also enhance its robustness against missing and noisy data [57].

## Conclusion

Using a ML approach in a prospective cohort of patients with DM, we identified important factors that predicted CV risk. If a patient is treated with ACEi or BB, is older and has/had a foot ulcer, this strongly predicts that she/he is at high risk of having overt CVD.

## Data Availability

The datasets used and/or analyzed during the current study are available from the corresponding author on reasonable request.

## Abbreviations

ACEi: Angiotensin converting enzyme inhibitor
AUC: Area under the curve
BB: Beta blocker
BMI: Body mass index
CAN: Cardiovascular autonomic neuropathy
CC: Correctly classified
CI: Confidence interval
CKD-EPI: Chronic Kidney Disease Epidemiology Collaboration
CV: Cardiovascular
DCA: Decision curve analysis
DM: Diabetes mellitus
DiCAN: Diabetic Cardiac Autonomic Neuropathy
DPN: Diabetic peripheral neuropathy
eGFR: Estimated glomerular filtration rate
EXCEL: Exenatide Study of Cardiovascular Event Lowering
HbA1c: Hemoglobin A1c
HR: Heart rate
ML: Machine learning
MLR: Multiple logistic regression
MNSI: Michigan Neuropathy Screening Instrument
RAND: Research and development
ROC: Receiver operating characteristic
SF-36: The 36-Item Shirt Form Survey
TECOS: Trial Evaluating Cardiovascular Outcomes with Sitagliptin
TIA: Transient ischemic attack
T1DM: Type 1 diabetes mellitus
T2DM: Type 2 diabetes mellitus
t-SNE: T-distributed stochastic neighbor embedding
UACR: Urinary albumin to creatin ratio
